# Inhibition of Biofilm Formation and Related Gene Expression of *Listeria monocytogenes* in Response to Four Natural Antimicrobial Compounds and Sodium Hypochlorite

**DOI:** 10.3389/fmicb.2020.617473

**Published:** 2021-01-14

**Authors:** Yunge Liu, Lina Wu, Jina Han, Pengcheng Dong, Xin Luo, Yimin Zhang, Lixian Zhu

**Affiliations:** ^1^ Lab of Beef Processing and Quality Control, College of Food Science and Engineering, Shandong Agricultural University, Tai’an, China; ^2^ National R&D Center for Beef Processing Technology, Tai’an, China; ^3^ Jiangsu Synergetic Innovation Center of Meat Production and Processing Quality and Safety Control, Nanjing, China

**Keywords:** *Listeria monocytogenes*, anti-biofilm mechanism, gene expression, quorum sensing, microscopic examinations

## Abstract

The aim of this study was to assess the efficacy of four natural antimicrobial compounds (cinnamaldehyde, eugenol, resveratrol and thymoquinone) plus a control chemical disinfectant (sodium hypochlorite) in inhibiting biofilm formation by *Listeria monocytogenes* CMCC54004 (Lm 54004) at a minimum inhibitory concentration (MIC) and sub-MICs. Crystal violet staining assay and microscopic examination were employed to investigate anti-biofilm effects of the evaluated compounds, and a real-time PCR assay was used to investigate the expression of critical genes by Lm 54004 biofilm. The results showed that five antimicrobial compounds inhibited Lm 54004 biofilm formation in a dose dependent way. Specifically, cinnamaldehyde and resveratrol showed better anti-biofilm effects at 1/4 × MIC, while sodium hypochlorite exhibited the lowest inhibitory rates. A swimming assay confirmed that natural compounds at sub-MICs suppressed Lm 54004 motility to a low degree. Supporting these findings, expression analysis showed that all four natural compounds at 1/4 × MIC significantly down-regulated quorum sensing genes (*agrA*, *agrC*, and *agrD*) rather than suppressing the motility- and flagella-associated genes (*degU*, *motB*, and *flaA*). This study revealed that sub-MICs of natural antimicrobial compounds reduced biofilm formation by suppressing the quorum sensing system rather than by inhibiting flagella formation.

## Introduction

*Listeria monocytogenes* (*L. monocytogenes*) is a Gram-positive food-borne pathogen. It is causes listeriosis with a high mortality rate (20–30%) among immunocompromised individuals (e.g., pregnant women, neonates, and the elderly; Gandra et al., 2019; Liu et al., 2020b). *L. monocytogenes* can survive for long periods under various harsh environmental conditions, such as high salt, low pH and refrigerated temperatures (Oloketuyi and Khan, 2017). The biofilm formation of *L. monocytogenes* is the main cause for its persistence and stress resistance in food processing environments (García-Gonzalo and Pagán, 2015). Biofilms are microbial communities that adhere to abiotic or biotic surfaces, which are surrounded by extracellular polymeric substances (Shi and Zhu, 2009). Once established, microorganisms in biofilms can enhance their resistance to antimicrobial agents, and thus, are more difficult to eradicate compared with planktonic cells (Jolivet-Gougeon and Bonnaure-Mallet, 2014; Chen et al., 2019). Consequently, *L. monocytogenes* cells in biofilms often cause recurrent contamination in food products, which enhances the food safety risks and leads to potential human health threats.

The molecular mechanism of biofilm formation by *L. monocytogenes* has been widely explored but is still not completely elucidated. At the initial period of biofilm formation, the swimming ability of flagella has been reported to be critical for bacteria to stick on the surface (Lemon et al., 2007). The genes associated with flagellar synthesis and motility involved in *L. monocytogenes* attachment, include *flaA*, *fliP*, *fliG*, *flgE*, *motA*, *motB*, *mogR*, and *degU* (Williams et al., 2005; Chang et al., 2012). Moreover, the quorum sensing (QS) system of *L. monocytogenes* (*agr* system) plays a critical role in its biofilm formation (Rieu et al., 2007). The locus *agr* in *L. monocytogenes* is composed of four genes, including *agrB*, *agrD*, *agrC*, and *agrA* (Zetzmann et al., 2016). In addition, the virulence regulator PrfA and the stress response regulator SigB also play important roles in *L. monocytogenes* biofilm development (Lemon et al., 2010; van der Veen and Abee, 2010). For this reason, exploring the efficacy of biofilm inhibitors against *L. monocytogenes* based on regulating key genes related with biofilm formation is a crucial and urgent task for the food industry.

Currently, natural antimicrobials have received strong interest as alternative agents of chemical antimicrobial drugs to inhibit biofilm formation (Xiang et al., 2019). Several studies have proved that active ingredients in essential oils are effective anti-biofilm agents against a variety of bacteria. Among them, phenylpropenes and phenolic compounds are consistently reported to be capable of inhibiting biofilm formation of pathogens. For instance, cinnamaldehyde and eugenol (phenylpropenes) were effective against the attachment of *Pseudomonas aeruginosa* and *Escherichia coli* O157:H7 by down-regulating curli genes (*casA* and *casB*) and Shiga-like toxin gene *stx2* (Kim et al., 2015). Likewise, previous studies also demonstrated that phenolic compounds prevented biofilm formation by pathogens, e.g., resveratrol (trans - 3,5,4′ - trihydroxystilbene) can disturb the expression of genes in the *agr* system related to QS and then inhibit biofilm formation of *Staphyloccocus aureus* (Qin et al., 2014). It is worth mentioning that the *agr* operon of *L. monocytogenes* is homologous to the *S. aureus* system (Zetzmann et al., 2016), which means that the QS inhibitors (QSIs, such as resveratrol) which acts on the *agr* system in *S. aureus* is probably a promising anti-QS agent for *L. monocytogenes*. In addition, anti-neoplastic agent, the constituent of Black cumin (*Nigella sativa*) thymoquinone (2-isopropyl-5-methyl-1,4-benzoquinone) was found to have significant anti-biofilm ability against Gram-negative (*E. coli* and *P. aeruginosa*) and Gram-positive bacteria (*Bacillus subtilis* and *S. aureus*; Goel and Mishra, 2018). A sub-inhibitory concentration of thymoquinone inhibited the production of AHL-regulated violacein pigment in *Chromobacterium violaceum*, meanwhile, RT-PCR assays also confirmed that thymoquinone down-regulated the transcription of the QS-relative gene *luxR* in *Cronobacter sakazakii* (Shi et al., 2017). Moreover, our previous study has investigated the effect of natural compounds on inhibiting the biofilm formation of wild *L. monocytogenes* (Lm 118) strain isolated from a beef processing plant (Liu et al., 2020a). However, the ability to form a biofilm was affected by the serotype and environmental conditions (Weiler et al., 2013), and the biofilm inhibition against a standard strain needs to be studied. Further, the inhibition mechanism of these compounds on *L. monocytogenes* should be more extensively elucidated.

This study is a continuation of our previous work (Liu et al., 2020a). Cinnamaldehyde, eugenol, resveratrol and thymoquinone were selected to compare the similarities and differences in the biofilm inhibition of *L. monocytogenes* CMCC54004 (Lm 54004), since they are different types of natural antibacterial compounds (phenylpropenes: cinnamaldehyde and eugenol; phenolic compound: resveratrol and benzoquinone compound: thymoquinone) which may help to elucidate the biofilm inhibitory mechanisms of this strain from different perspectives. Moreover, previous studies have identified numerous genes associated with *L. monocytogenes* biofilm formation at 37°C (Upadhyay et al., 2013; Miao et al., 2019), rather than the lower temperatures associated with ambient indoor room settings. In this study, in order to achieve the effective biofilm development, 25°C was used as the incubation temperature (Liu et al., 2020a). To determine the ability of natural antimicrobial compounds to prevent biofilm synthesis, sub-minimal inhibitory concentrations (concentrations that exhibited no significant inhibitory effect on the growth of planktonic bacteria) of the compounds were used. Firstly, the anti-biofilm activity of these four natural compounds and sodium hypochlorite (disinfectant frequently used in the food processing facilities) were evaluated against Lm 54004 on polystyrene surfaces. Polystyrene was chosen as it is one of the most utilized materials in the food industry. Secondly, scanning electron microscopy (SEM) and confocal laser scanning microscopy (CLSM) were used to evaluate the effects of antimicrobial compounds on the biofilm architecture and cellular viability of Lm 54004. Moreover, the effects of natural antimicrobial compounds on the expression of critical biofilm-associated genes (*agrA*, *agrC*, *agrD*, *prfA*, *sigB*, *relA*, *inlA*, *degU*, *motB*, and *flaA*) of Lm 54004 were measured by quantitative reverse transcription PCR (RT-qPCR).

## Materials and Methods

### Bacterial Strains and Preparation of Inoculum

The bacterial strain *L. monocytogenes* CMCC 54004 (Lm 54004, serotype 1/2a) was purchased from the China National Center for Medical Culture Collections (CMCC), source from Czech Institute of Epidemiology and Microbiology. The strain was stored at −80°C in the brain heart infusion (BHI; Beijing Land Bridge Technology, China) with 25% (v/v) glycerol. The strain was activated by transferring 0.2 ml of the frozen culture into 20 ml of BHI and incubating at 37°C for 18 h with two consecutive transfers.

Four natural antimicrobial compounds: cinnamaldehyde, eugenol, resveratrol, and thymoquinone were obtained from Macklin, China; Solarbio, China and Yuanyeshengwu, China respectively. The control compound, sodium hypochlorite (10% active chlorine), was obtained from Sinopharm Chemical Reagent, China. The purity of all the compounds was above 98%. The four natural compounds were diluted with 1% dimethyl sulfoxide (DMSO) in BHI, with 1% DMSO shown not to exhibit an adverse effect on the growth of *L. monocytogenes* (Fan et al., 2018).

### Minimal Inhibitory Concentration Determination

Minimal inhibitory concentrations (MICs) of the above-mentioned four natural antimicrobial compounds and sodium hypochlorite were determined by the microdilution method, as described by the Clinical and Laboratory Standards Institute (CLSI) with some modification. Briefly, each of the compounds was diluted in a 96-well microtiter plate, final concentrations of cinnamaldehyde and eugenol ranging from 20 to 2,560 μg/ml, resveratrol and thymoquinone between 12.5 and 400 μg/ml and sodium hypochlorite from 195 to 6,250 ppm. The final tested concentration of Lm 54004 was 5 × 10^5^ CFU/ml. Broth only (microtiter wells containing uninoculated BHI medium) was used as negative control. The plate was incubated at 37°C for 24 h under static conditions. The MIC was defined as the lowest concentration of compounds that inhibited visible bacterial growth.

### Sub-MICs Determination

Sub-MICs of the above-mentioned antimicrobial compounds against Lm 54004 were assessed using a growth curve analysis as previously described (Fan et al., 2018). The bacteria was grown overnight in BHI, and after that, the bacterial suspension was adjusted to a cell concentration of 5 × 10^5^ CFU/ml. Then it was inoculated into BHI with or without compounds at 1/32 × MIC, 1/16 × MIC, 1/8 × MIC, 1/4 × MIC, 1/2 × MIC, MIC, 2 × MIC and 4 × MIC. The cultures were incubated at 37°C for 24 h, and the assay optical density (OD) at 600 nm was determined at 1 h intervals.

### Effects of Antimicrobial Compounds on Cell Motility

Swimming and swarming assays were conducted using a semisolid motility agar as previously described with some modifications (Li et al., 2018). Briefly, 3 μl of an overnight culture of Lm 54004 was inoculated at the center of swimming (10 g/L tryptone, 5 g/L NaCl and 0.3% agar) and swarming (25 g/L LB, 0.5 g/L glucose, 0.5% agar) plates containing different concentrations (1/8 × MIC, 1/4 × MIC and MIC) of four natural antimicrobial compounds and sodium hypochlorite. BHI was set as the negative control. After incubation for 48 h at 25°C, the diameter (mm) of the motility zones was measured.

### Effects of Antimicrobial Compounds on Biofilm Formation

Inhibition of biofilm formation by above mentioned antimicrobial compounds was studied using the crystal violet assay (Fan et al., 2018). Briefly, 100 μl BHI supplemented with different concentrations (1/8 × MIC, 1/4 × MIC, 1/2 × MIC and MIC) of antimicrobial compounds was added in 96-well microtiter plates. Subsequently, bacterial suspension of Lm 54004 (100 μl, 1 × 10^6^ CFU/ml) was inoculated into wells. Bacterial cultures without the addition of antimicrobials were used as a positive control. Broth only (microtiter wells containing 200 μl of uninoculated BHI medium) was used as the negative control. After 72 h of incubation at 25°C, the wells were washed with sterile distilled water three times to remove the planktonic bacteria. Then, 200 μl of 0.25% (w/v) crystal violet was added to each well and stained for 30 min at room temperature. Next, the crystal violet solution was removed and 200 μl of 95% (v/v) ethanol was added to solubilize the stain, and absorbance was measured spectrophotometrically at 570 nm. The inhibitory rates were then calculated using the following formula: Inhibitory rate (%) = [1 − OD_570nm_ (Sample) /OD_570nm_ (positive control)] × 100. The whole experiment was replicated three times independently.

### Effects of Antimicrobial Compounds on Biofilm Metabolic Activity

Bacterial viability was analyzed using the Cell Counting Kit-8 (CCK-8, 7Sea Biotech, Shanghai, China) as previously described (Yu et al., 2017). Biofilms were grown as stated above with BHI containing antimicrobial compounds (1/8 × MIC, 1/4 × MIC, and MIC) in 96-well microtiter plates. The negative controls contained only BHI. After 72 h of incubation at 25°C, the supernatant was discarded and replaced with 100 μl sterile PBS and 10 μl CCK-8 dye solution. Then plates were incubated for 4 h at 25°C. The absorbance was then measured at 450 nm using the microplate reader (SpectraMax M5, Molecular Devices, United States). All experiments were repeated three times independently.

### Analysis of Biofilms by Scanning Electron Microscopy and Confocal Laser-Scanning Microscopy

For the SEM analysis, individual polystyrene (PS) coupons (2 mm thick and 10 mm in diameter) were placed horizontal in 48-well polystyrene microtiter plates. Subsequently, 300 μl of BHI containing antimicrobial compounds (1/8 × MIC, 1/4 × MIC and MIC) was added into wells, respectively. The negative control contained only BHI was also visualized to determine the normal architecture of the biofilm. Next, a 300 μl bacterial suspension of Lm 54004 was inoculated into culture (final tested concentration of bacteria was 5 × 10^5^ CFU/ml). The plates were incubated statically at 25°C for 72 h to favor biofilm formation. After the incubation, the chips were gently washed with sterile PBS and immersed in 2.5% glutaraldehyde at 4°C for 24 h. After washing thrice with PBS, the cultures were then dehydrated in a gradient alcohol concentration (50, 70, 80, 90, and 100%) for 10 min at each concentration. After critical point drying with liquid carbon dioxide (CO_2_), and gold coating, the samples were examined using a SU8020 scanning electron microscope (Hitachi, Tokyo, Japan).

For the CLSM analysis, Lm 54004 was inoculated into BHI with antimicrobial compounds (1/8 × MIC, 1/4 × MIC, and MIC) in cell culture dishes (35 mm × 10 mm, Sigma, United States). Biofilms not exposed to antimicrobial compounds (negative control) was also visualized to determine the normal architecture of the biofilm. The final concentration of bacteria was 5 × 10^5^ CFU/ml. After 72 h of incubation at 25°C, the bacteria were gently washed with sterile PBS three times. Then the biofilms were stained with the LIVE/DEAD BacLight kit L-7012 (Molecular Probes, United States) for 30 min in the dark. The kit included Syto 9 which labels all bacteria with intact membranes, and propidium iodide which only penetrates and stains cells with damaged membranes. Biofilm samples were imaged under a confocal laser microscope (LSM 880, Zeiss, Germany) using a 63 × oil immersion objective lens with a 488-nm argon laser, and the emitted fluorescence was recorded within the range of 480–500 nm to collect Syto 9 emission fluorescence and 490–635 nm to collect propidium iodide-emitted fluorescence. Three-dimensional projections were reconstructed from z-stacks using the easy 3D function of the ZEN Blue Lite 2_3 software.

### Quantitative Real-Time PCR

RT-qPCR was used to evaluate the effect of antimicrobial compounds on the expression of genes associated with biofilm formation. Firstly, Lm 54004 was inoculated into BHI with or without antimicrobial compounds (1/4 × MIC) in cell culture dishes, incubation at 25°C for 72 h. Cell culture dishes were washed with sterile PBS for three times. Total RNA of biofilms in cell culture dishes were extracted by using MiniBEAT Universal RNA Extraction Kit (Takara, China). Next, total RNA was reverse transcribed into cDNA by using Takara PrimeScript™ RT Reagent Kit (Takara, Beijing, China). SYBR^®^ Premix Ex *Taq*™ (Takara, China) were applied in RT-PCR to quantify gene expression. The primers for evaluated genes (Table 1) were previously published (Upadhyay et al., 2013; Du et al., 2018) and synthesized by BioSsune Co., Ltd. (Shanghai, China). 16S rRNA was selected as an internal standard. Quantification of mRNA was performed with a real-time PCR system (CFX96, Bio-Rad, United States) with CFX 96 (Bio-Rad, United States). The 2^-ΔΔ*Ct*^ method was used to analyze the relative gene expression obtained according to the melting curve (Livak and Schmittgen, 2001).

**Table 1:** List of primers used in this study.

Gene name	Primer name	Primer sequence (5'-3')
*16 s r*RNA	*16S*-F	ACCGTCAAGGGACAAGCA
	*16S*-R	GGGAGGCAGCAGTAGGGA
*agrA*	*agrA*-F	GCAGCCGGACATGAATGG
	*agrA*-R	AACCACGCGGATCAAACTTC
*agrC*	*agrC*-F	GGGGTCAATCGCAGGTTTTG
	*agrC*-R	CTTTAAGTTCGTTGGTTGCCGTA
*agrD*	*agrD*-F	AAATCAGTTGGTAAATTCCTTTCTAG
	*agrD*-R	AATGGACTTTTTGGTTCGTATACA
*relA*	*relA*-F	TGCGATGCCGAAGTCGAATA
	*relA*-R	GCAACCCCGTATTCAGCGAT
*sigB*	*sigB*-F	TGGATTGCCGCTTACCAAGAA
	*sigB*-R	TCGGGCGATGGACTCTACTA
*prfA*	*prfA*-F	TGAGCAAGAATCTTACGCACTTTT
	*prfA*-R	GCTAGGCTGTATGAAACTTGTTTTTG
*inlA*	*inlA*-F	ACTTGGCAGTGGAGTATGGA
	*inlA*-R	CTGAAGCGTCGTAACTTGGTC
*degU*	*degU*-F	ACGCATAGAGAGTGCGAGGTATT
	*degU*-R	CCCAATTCCGCGGTTACTT
*flaA*	*flaA*-F	GGCTGCTGAAATGTCCGAAA
	*flaA*-R	TGCGGTGTTTGGTTTGCTTG
*motB*	*motB*-F	AATCGCCAAAGAAATCGGCG
	*motB*-R	CGCCGGGGTTTACTTCACTA

### Statistical Analyses

Triplicate independent experiments were conducted for each of the above assays. The MIXED procedure (Statistical Analysis System, SAS, version 9.0) was applied to analyze the biofilm inhibitory rate while the fixed factors were concentration, compound type and their interaction, and the random factor was experiment replication. The Tukey Multiple Comparison Test was performed to determine the influence of concentration of each compound on motility ability and biofilm metabolic activity. The results of relative gene expressions were analyzed by a *t*-test using SPSS version 18.0 to compare the difference between the experimental groups and the control group. Differences were considered statistically different at *p* < 0.05.

## Results

### Minimum Inhibitory Concentrations

All compounds inhibited the growth of Lm 54004, and the MICs for cinnamaldehyde, eugenol, resveratrol, thymoquinone, and sodium hypochlorite were 640 μg/ml, 1,280 μg/ml, 400 μg/ml, 50 μg/ml and 1560 ppm, respectively.

### Growth Curves in Sub-MICs

**Figure 1:**
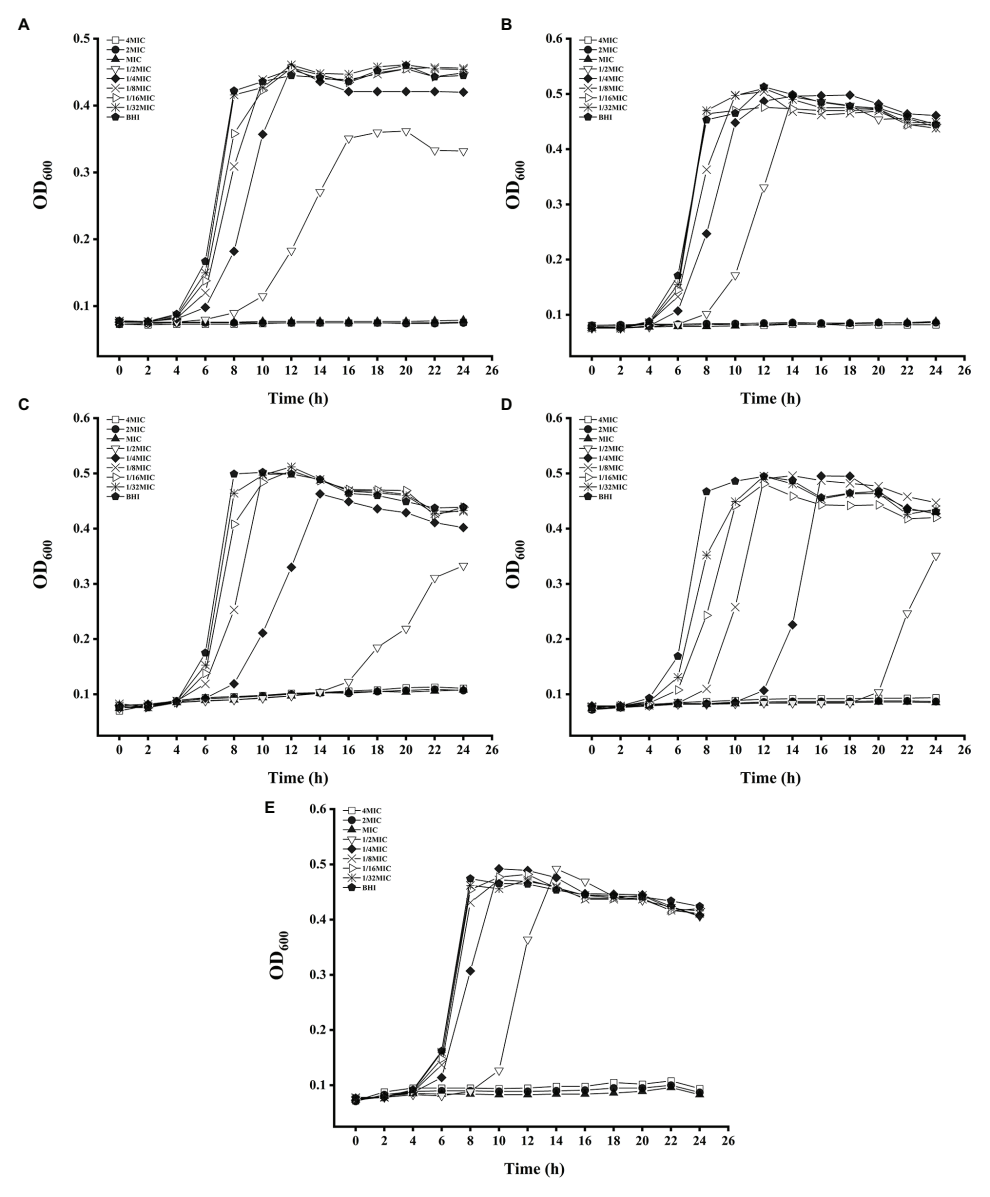
Growth curves of *Listeria monocytogenes* CMCC 54004 incubated with five antimicrobials compounds for 24 h at 37°C. **(A)**: cinnamaldehyde, **(B)**: eugenol, **(C)**: resveratrol, **(D)**: thymoquinone, **(E)**: sodium hypochlorite.

Our results showed that the growth of planktonic bacteria was totally inhibited by compounds at MIC – 4 × MIC, while 1/32 × MIC – 1/4 × MIC of five compounds all exhibited no obvious impacts on the concentration of Lm 54004 at stationary phase (Figure 1). Thus, the concentration of 1/8 × MIC and 1/4 × MIC were chosen as the sub-MICs in this study for the following experiments. Specifically, as follows: Cinnamaldehyde (80 μg/ml and 160 μg/ml), eugenol (160 μg/ml and 320 μg/ml), resveratrol (50 μg/ml and 100 μg/ml), thymoquinone (6.25 μg/ml and 12.5 μg/ml) and sodium hypochlorite (195 and 390 ppm).

### Cell Motility

**Figure 2:**
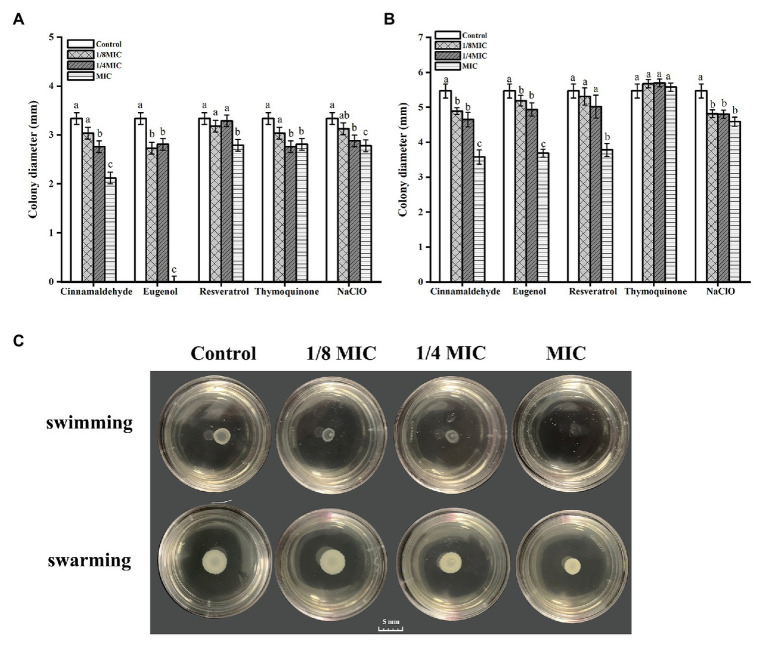
Efficacy of five antimicrobials compounds in inhibiting motility ability of *Listeria monocytogenes* CMCC 54004 grown at 25°C. **(A)**: swimming ability, **(B)**: swarming ability, **(C)**: representative plate images of swim and swarm rings of Eugenol (images of all compounds was shown in the attachment). a-c Indicate the same compound treatments at different concentrations with different letters are significantly different (*p* < 0.05). Mean values of three independent experiments and standard error are shown.

Assays for swimming and swarming motility on semi-solid plates showed that Lm 54004 diffused on the agar, and after 48 h of incubation at 25°C, colony sizes were 3.34 mm and 5.47 mm, respectively (Figure 2, control). For swimming motility, at 1/8 × MIC only eugenol significantly inhibited swimming motility of Lm 54004, while other compounds had no inhibitory effects on the colony diameters compared with the control group (Figure 2A). Treatment with 1/4 × MIC of cinnamaldehyde, eugenol, thymoquinone and sodium hypochlorite all significantly reduced the swimming motility of Lm 54004, but resveratrol at 1/4 × MIC had no inhibitory effects (Figure 2A). After treatment with MIC of all evaluated compounds, Lm 54004 showed a significantly low swimming motility (*p* < 0.05), with a colony size of below 3 mm (Figure 2A). For swarming motility, thymoquinone exerted no inhibitory effect at all test concentrations, resveratrol only inhibited it at MIC, cinnamaldehyde and eugenol significantly inhibited it at 1/4 × MIC and 1/8 × MIC (Figure 2B).

### Biofilm Inhibitory Rate on the Polystyrene Surface

Results on the biofilm inhibitory rate of Lm 54004 treated by different antimicrobial compounds are shown in Table 2. Four natural antimicrobial compounds all exerted strong biofilm inhibition, even at sub-MICs, where biofilm inhibitory rates on polystyrene microplate ranged from 37.6 to 53.8% at the presence of each compound at 1/8 × MIC, with a higher inhibitory rate of 46.4–68.0% evident as the concentration of compounds increased to 1/4 × MIC. Moreover, thymoquinone and eugenol showed a significantly lower biofilm inhibitory effect compared to cinnamaldehyde and resveratrol at 1/4 × MICs. The biofilm inhibitory effect of the common chemical disinfectant sodium hypochlorite was 34.5% (1/4 × MIC) and 30.8% (1/8 × MIC), which were significantly lower than the four natural antimicrobial compounds.

**Table 2:** Biofilm inhibitory rate of *Listeria monocytogenes* CMCC 54004 on the polystyrene surface in different compounds and concentrations (25°C, 72 h).

Concentration	Biofilm inhibitory rate (%)					SE^e^
	Cinnamaldehyde	Eugenol	Resveratrol	Thymoquinone	Sodium hypochlorite	
1 × MIC	75.35^ai^	74.57^ai^	77.59^ai^	71.41^ai^	60.13^bi^	2.94
1/4 × MIC	66.11^aj^	51.09^bj^	68.01^aj^	46.66^bj^	34.46^cj^	
1/8 × MIC	40.88^bck^	44.48^bj^	53.76^ak^	37.62^bck^	30.83^cj^	

^a-c^Means with different letters in the same row indicate significant differences (*p* < 0.05).

^i-k^Means with different letters in the same column indicate significant differences (*p* < 0.05).

^e^SE = standard error.

### Biofilm Metabolic Activity

**Figure 3:**
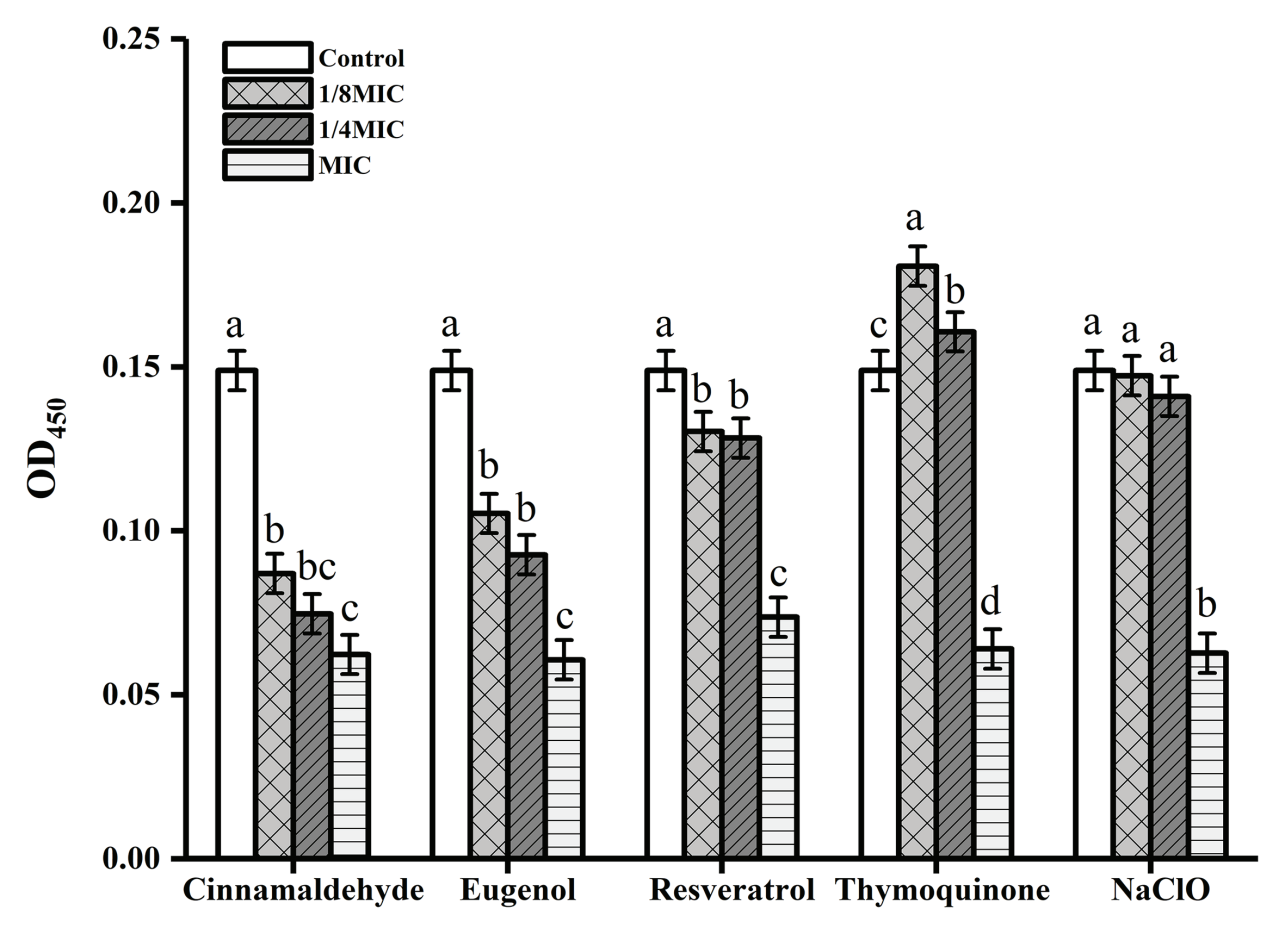
Efficacy of five antimicrobials compounds in inhibiting biofilm metabolic activity of *Listeria monocytogenes* CMCC 54004 grown at 25°C. a-d Indicate the same compound treatments at different concentrations with different letters are significantly different (*p* < 0.05). Mean values of three independent experiments and standard error are shown.

In this study, the Cell Counting Kit-8 (CCK-8) assay was used to reveal the metabolic status of the cells in biofilms (Figure 3). Our results of the CCK-8 assay confirmed that all evaluated antimicrobial compounds significantly inhibited metabolic activity of the biofilms formed by Lm 54004 (*p* < 0.05) at MIC (Figure 3). Sub-MICs (1/4 × MIC and 1/8 × MIC) of cinnamaldehyde, eugenol and resveratrol also showed significant impacts on reducing the Lm 54004 cell viability in the biofilms (Figure 3). However, the biofilm metabolic activity was significantly higher in thymoquinone sub-MICs (1/4 × MIC and 1/8 × MIC) treatment groups compared with that in the control group (Figure 3). Moreover, the control compound sodium hypochlorite at sub-MICs had no inhibitory effect on the cell viability.

### SEM and CLSM Observation

**Figure 4:**
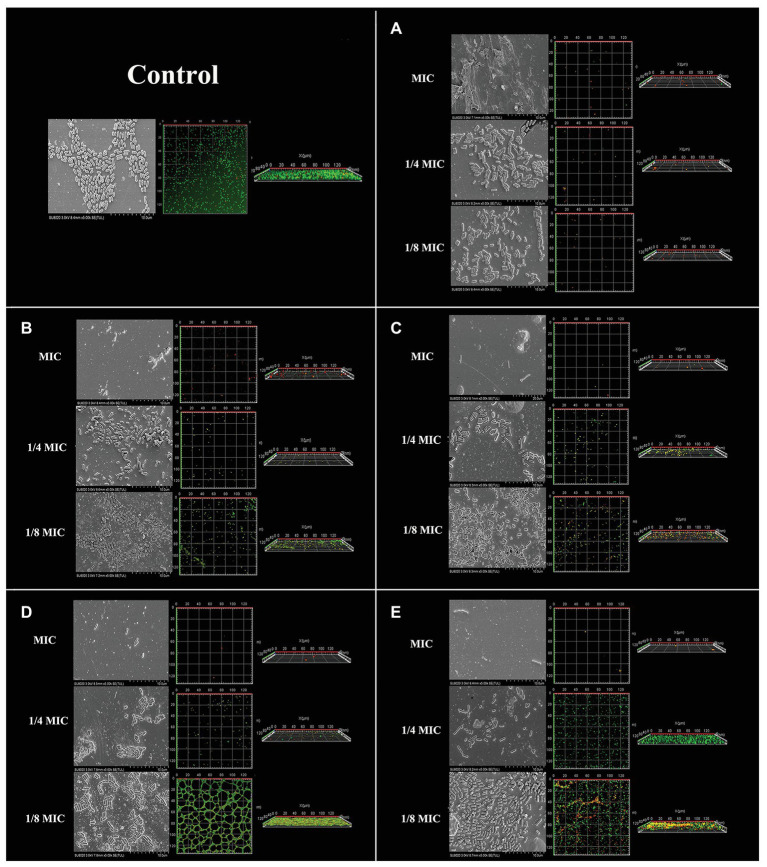
Scanning electron microscopy (SEM) and confocal laser-scanning microscopy (CLSM) of *Listeria monocytogenes* CMCC 54004 biofilms formed in the presence of five antimicrobials compounds. **(A)**: cinnamaldehyde, **(B)**: eugenol, **(C)**: resveratrol, **(D)**: thymoquinone, **(E)**: sodium hypochlorite.

The CLSM and SEM analysis showed that Lm 54004 biofilms revealed dose-related changes (Figure 4). The CLSM showed that all five antimicrobial compounds considerably reduced biofilm formation, with visible dose-dependent alterations and decreased cellular density in the three-dimensional structural organization of cells in the biofilm. Moreover, it is clear that the four natural antimicrobial compounds decreased the cellular density even at 1/4 × MIC (Figures 4A–D), but sodium hypochlorite had less inhibitory effect (Figure 4E). These changes were also in accord with the increasing numbers of dead bacteria (in red) seen by LIVE/DEAD staining. In the SEM images, the observation of cellular density was consistent with the CLSM results. The control cells in SEM images appeared intact, plump and typically rod-shaped with a smooth exterior, however when exposed to antimicrobial compounds, the cell damage could be visualized directly, where cells distorted from their normal shape or even ruptured, and more extracellular matrix could be observed.

### Effect of Antimicrobial Compounds on Expression of Genes Critical for Biofilm Formation

**Figure 5:**
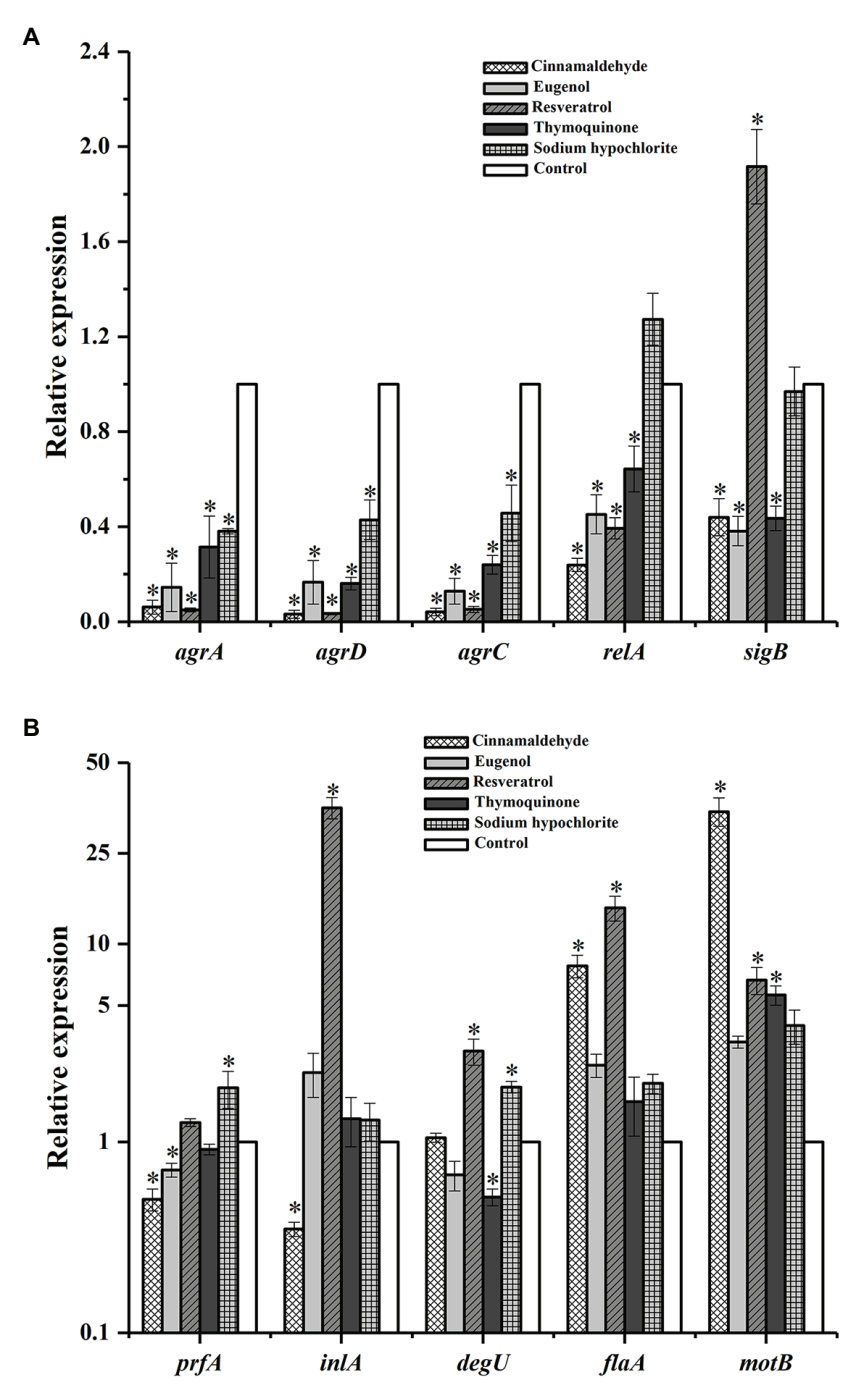
Relative gene expression of *Listeria monocytogenes* CMCC 54004 in response to five antimicrobials compounds (at 1/4 × MIC). **(A)**: relative expression of genes between 0 and 2.4, **(B)**: relative expression of genes between 0.1 and 50. ^*^Indicates *p* < 0.05 vs. the control group. Mean values of three independent experiments and SE are shown.

Quantitative reverse transcription PCR was used to analyze the transcriptional response of some genes related to biofilm and virulence in 1/4 × MIC antimicrobial-treated cells vs. untreated bacteria. The expression of the genes tested were compound dependent. As compared with the untreated control, four natural antimicrobial compounds substantially down-regulated the expression of quorum-sensing genes (*agrA*, *agrC*, and *agrD*) and starvation responses regulation gene *relA* (Figure 5A). Likewise, natural antimicrobial compounds also down-regulated the expression of *sigB* (global regulator of the stress response) except of the resveratrol (Figure 5A). As shown in Figure 5B, cinnamaldehyde and eugenol significantly down-regulated the transcription of *prfA* (the major regulator of *L. monocytogenes* virulence factors), while the gene was not obviously affected by resveratrol and thymoquinone. The virulence gene *inlA* (encodes internalin) was significantly suppressed (*p* < 0.05) by cinnamaldehyde, while not obviously affecting the expression of *inlA* (*p* > 0.05) when exposed to eugenol and thymoquinone, but resveratrol up-regulated the expression of *inlA*. Moreover, the expression of motility- and flagella-associated genes (*degU*, *flaA*, and *motB*) were all significantly up-regulated (*p* < 0.05) by resveratrol, and cinnamaldehyde induced the expression of *flaA* and *motB*, while eugenol exerted no effect on the expression of these genes and thymoquinone down-regulated the transcription of *degU*. Meanwhile, we found that the disinfectant sodium hypochlorite only down-regulated the expression of *agrA* and *agrD*, and no suppressive effect was found on other genes.

## Discussion

In the present study, we selected the common chemical disinfectant sodium hypochlorite as the control compound. Rodriguez-Melcon et al. (2019) reported that MIC or 1.5 × MIC of sodium hypochlorite notably reduced the biovolume and cellular viability of *L. monocytogenes* biofilms. Similarly, our results showed that sodium hypochlorite at MIC exhibited a strong anti-biofilm activity against Lm 54004 (Table 2). However, compared to the natural antimicrobial compounds, sodium hypochlorite also showed significantly lower inhibitory effects on Lm 54004 biofilm both at MIC and 1/4 × MIC. This finding is consistent with a previous report that the biofilm elimination effect of essential oils (cinnamon, marjoram, and thyme) was in most cases better compared to sodium hypochlorite (Vidács et al., 2018). The corresponding results were also revealed by the RT-qPCR assays in this study (Figure 5), that sodium hypochlorite at 1/4 × MIC had less suppressive effects on biofilm-related genes compared to the four natural antimicrobial compounds.

As mentioned above, biofilm inhibitory effects of the four natural antimicrobial compounds on Lm 54004 are different (Table 2). Miao et al. (2019) proved that thymoquinone effectively reduced biofilm biomass of *L. monocytogenes* ATCC19115 at sub-MICs. In the current study, we also found that thymoquinone was an effective anti-biofilm agent compared to the sodium hypochlorite. However, thymoquinone showed a lower biofilm inhibition rate at sub-MICs compared to the phenylpropenes (cinnamaldehyde and eugenol) and the phenolic compound (resveratrol) against Lm 54004. In addition, the results were confirmed by the biofilm metabolic activity assay, that thymoquinone was the least compound to inhibit the biofilm metabolic activity of Lm 54004 at sub-MICs (Figure 3).

The gene *prfA* is a global regulator which positively regulates virulence genes (like *inlA*) in *L. monocytogenes* biofilms and *sigB* is the global regulator of the stress response and is also closely related to virulence (Lemon et al., 2010; Vazquez-Armenta et al., 2020). Moreover, *relA* regulated the starvation responses in *L. monocytogenes*, which is an essential gene for cells survival in nutrient deficiency conditions (Kocot and Olszewska, 2017). Studies suggest that virulence genes are closely related to the biofilm development in *L. monocytogenes* (Vazquez-Armenta et al., 2020) and Sivaranjani et al. (2016) found that morin inhibited biofilm formation while interrupting the secretion of virulence determinant Listeriolysin O (LLO). In the present study, 1/4 × MIC of all four natural compounds down-regulated the expression of *relA*. Cinnamaldehyde and eugenol were more effective in suppressing the *prfA* and *sigB*, and thymoquinone only down-regulated the transcription of *sigB* (Figure 5). These results are consistent with the biofilm inhibition assay, which indicates that the suppression of *prfA* and *sigB* was a critical reason for natural compounds to inhibit the formation of *L. monocytogenes* biofilm. However, resveratrol significantly induced the expression of *sigB*, and had no suppressive effects on *prfA*. Furthermore, resveratrol also up-regulated the virulence gene *inlA* while other natural compounds had no effects on this gene. These results suggest that resveratrol increased the stress responses of the *L. monocytogenes* cells to resist the external harsh environment. A similar result was found by Huang et al. (2020), such that the photodynamic inactivation treatment up-regulated the expression of *prfA* while it markedly reduced the adhesion ability of the biofilms of *L. monocytogenes*. Therefore, biofilm inhibition is a complex process, suggesting that the biofilm inhibitory pathway of various antimicrobial compounds differs. Further investigation is needed to explore the biofilm inhibitory pathway, based on the global regulators (*prfA* and *sigB*), of different antimicrobial compounds against *L. monocytogenes.*

Moreover, previous reports demonstrated that the motility ability of flagella was important in early biofilm formation (Bonsaglia et al., 2014). In this study, 1/4 × MIC of cinnamaldehyde, eugenol and thymoquinone inhibited the swimming motility of Lm 54004 compared to the control (Figure 2A), although the colony diameters did not change much (within 0.6 mm), responding with inhibitory rates all below 18%. Additionally, resveratrol at 1/4 × MIC had no inhibitory effects on the swimming and swarming motility of Lm 54004 cells. *degU* is the response regulator in *L. monocytogenes* which is involved in the flagellin expression and motility genes (Williams et al., 2005). The results of RT-qPCR showed that only thymoquinone was effective in down-regulating the expression of *degU*, while the other three natural compounds had no suppressive effects on this gene. *motB* is the gene that encodes for the motility protein involved in flagellar motor rotation, and *flaA* is a flagella-associated gene (Casey et al., 2014). The expression of these two motility-associated genes were both not suppressed or induced by the four natural compounds at 1/4 × MIC compared to the control, but the biofilm formation was significantly reduced at this concentration when treated by these compounds. As showed in the previous studies, Upadhyay et al. (2013) and Miao et al. (2019) found that cinnamaldehyde, eugenol, and thymoquinone effectively inhibited the motility of *L. monocytogenes* and significantly down-regulated the expression of *flaA*. The reason for these different findings as reported is probably due to the temperature (25°C) applied in the current study, and the bacterial strain (or serotype) we used was different to those in the studies of Upadhyay et al. (2013) and Miao et al. (2019). According to Bonsaglia et al. (2014), temperature is an important factor which may affect the formation of flagella in *L. monocytogenes*. The results confirmed that sub-MIC of natural antimicrobial compounds which reduced biofilm formation might not inhibit flagella formation.

It has been suggested that the QS system of *L. monocytogenes* plays a critical role in its biofilm formation (Rieu et al., 2007; Brackman and Coenye, 2015). QS is an intercellular communication system by which bacteria can coordinate their population density and control a variety of physiological processes (Solano et al., 2014). In *L. monocytogenes*, the QS is regulated by the Agr system for intraspecies communication (Skandamis and Nychas, 2012; Kocot and Olszewska, 2017). Previous study showed that mutations of *agrA* and *agrD* genes displayed significantly reduced biofilm formation of *L. monocytogenes* (Rieu et al., 2007; Riedel et al., 2009). In this study, the expression of three QS-associated genes (*agrA*, *agrC*, and *agrD*) were all significantly suppressed by four natural antimicrobial compounds at 1/4 × MIC (Figure 5). The research of Du et al. (2018) reported similar results and found that low concentrations of epigallocatechin gallate inhibited biofilm formation by suppressing the QS system. Based on previous studies a number of different ways to inhibit the QS signaling molecules have been proposed, such as signal binding, degradation of the signaling molecules, competitive inhibition and genetic regulation systems (Sankar Ganesh and Ravishankar Rai, 2018). For example, Jakobsen et al. (2012) found that isothiocyanate produced from horseradish inhibited the expression of the lasB-gfp fusion, which compete with AHL signaling molecules (in Gram-negative bacteria) of regulator proteins. In this study, four natural compounds were all shown to block Agr QS systems of *L. monocytogenes*, probably by degrading signal receptors or secreting signal degrading enzymes and signal mimics, etc. Therefore, further research should be undertaken to investigate the direct mechanism of cinnamaldehyde, eugenol, resveratrol and thymoquinone on the QS system of *L. monocytogenes*. In summary, natural antimicrobial compounds at low concentrations were more likely to suppress the QS-associated genes to inhibit the biofilm formation of *L. monocytogenes*. For this reason, QS inhibition is a good point to study the anti-biofilm mechanism of natural antimicrobial compounds on *L. monocytogenes*.

## Conclusion

This study showed that sub-MIC of cinnamaldehyde, eugenol, resveratrol, and thymoquinone were all efficient at inhibiting Lm 54004 biofilms, of which, cinnamaldehyde and resveratrol showed better anti-biofilm effects. Meanwhile, the transcriptional results showed that sub-MIC of natural antimicrobial compounds reduced biofilm formation by suppressing the QS system rather than by inhibiting flagella formation. In addition, the biofilm inhibitory pathway of different antimicrobial compounds differs, which needs further exploration. The findings of present study suggest that low concentrations of natural compounds can serve as potential antimicrobials in controlling biofilm of *L. monocytogenes* in the food industry. The effective application of these compounds in industry could be achieved by using them in combination with chemical and physical disinfection methods commonly used in food processing, such as organic acids, sodium hypochlorite, or UV light, high temperature or high pressure.

## Data Availability Statement

The original contributions presented in the study are included in the article/Supplementary Material, further inquiries can be directed to the corresponding authors.

## Author Contributions

YL: conceptualization, investigation, formal analysis, data curation, and writing - original draft. LW: conceptualization, investigation, and resources. JH: conceptualization, resources, and methodology. PD: conceptualization, investigation, resources, and methodology. XL: conceptualization, writing - review and editing, and supervision. YZ: conceptualization, writing - reviewing and editing, and funding acquisition. LZ: conceptualization, supervision, funding acquisition, and methodology. All authors contributed to the article and approved the submitted version.

### Conflict of Interest

The authors declare that the research was conducted in the absence of any commercial or financial relationships that could be construed as a potential conflict of interest.

## Supplementary Material

Supplementary Figure 1 Plate images of five antimicrobials compounds in inhibiting motility ability of *Listeria monocytogenes* CMCC 54004 grown at 25°C. **(A)**: cinnamaldehyde, **(B)**: eugenol, **(C)**: resveratrol, **(D)**: thymoquinone, **(E)**: sodium hypochlorite.
