# Supplementary figures and images for: Inhibition of Biofilm Formation and Related Gene Expression of Listeria monocytogenes in Response to Four Natural Antimicrobial Compounds and Sodium Hypochlorite
Source: Front Microbiol. 2021 Jan 14;11:617473. doi: 10.3389/fmicb.2020.617473 (PMC7840700; doi:10.3389/fmicb.2020.617473)

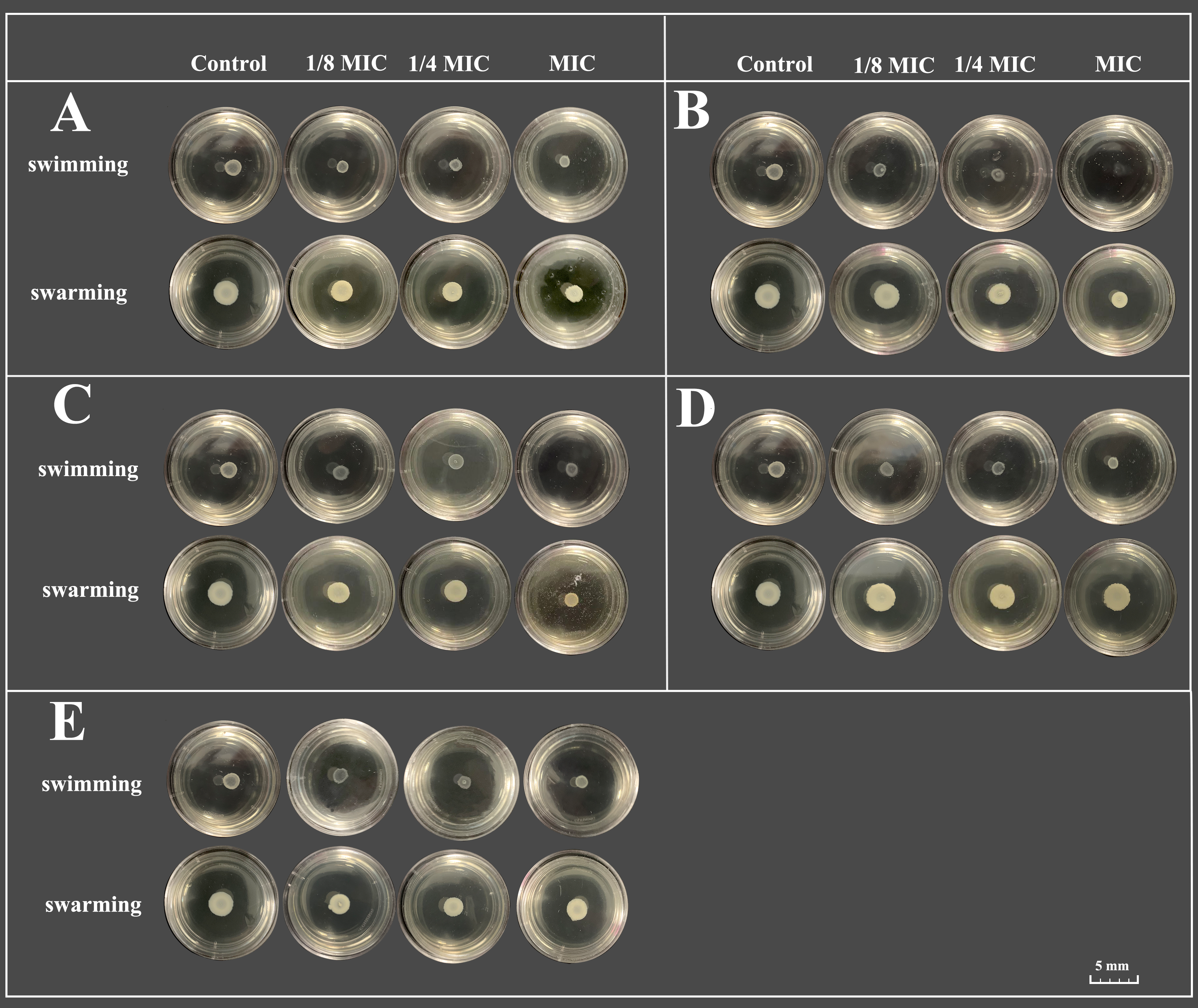

Supplement: Supplementary file 1 [file Image_1.tif]
